# Optimizing Genomic-Enabled Prediction in Small-Scale Maize Hybrid Breeding Programs: A Roadmap Review

**DOI:** 10.3389/fpls.2021.658267

**Published:** 2021-07-01

**Authors:** Roberto Fritsche-Neto, Giovanni Galli, Karina Lima Reis Borges, Germano Costa-Neto, Filipe Couto Alves, Felipe Sabadin, Danilo Hottis Lyra, Pedro Patric Pinho Morais, Luciano Rogério Braatz de Andrade, Italo Granato, Jose Crossa

**Affiliations:** ^1^Laboratory of Allogamous Plant Breeding, Genetics Department, Luiz de Queiroz College of Agriculture, University of São Paulo, Piracicaba, Brazil; ^2^Department of Epidemiology and Biostatistics, Michigan State University, East Lansing, MI, United States; ^3^Department of Computational and Analytical Sciences, Rothamsted Research, Harpenden, United Kingdom; ^4^Department of Agronomy, Federal University of Viçosa, Viçosa, Brazil; ^5^Brazilian Agricultural Research Corporation (EMBRAPA), Cassava and Fruits, Cruz das Almas, Brazil; ^6^Laboratoire d'Ecophysiologie des Plantes sous Stress Environnementaux (LEPSE), Institut National de la Recherche Agronomique (INRA), Univ. Montpellier, SupAgro, Montpellier, France; ^7^Biometrics and Statistics Unit, International Maize and Wheat Improvement Center (CIMMYT), Carretera México - Veracruz, Texcoco, Mexico; ^8^Colegio de Posgraduado, Montecillo, Mexico

**Keywords:** accuracy, quantitative genomics, R packages, genomic selection, breeding schemes

## Abstract

The usefulness of genomic prediction (GP) for many animal and plant breeding programs has been highlighted for many studies in the last 20 years. In maize breeding programs, mostly dedicated to delivering more highly adapted and productive hybrids, this approach has been proved successful for both large- and small-scale breeding programs worldwide. Here, we present some of the strategies developed to improve the accuracy of GP in tropical maize, focusing on its use under low budget and small-scale conditions achieved for most of the hybrid breeding programs in developing countries. We highlight the most important outcomes obtained by the University of São Paulo (USP, Brazil) and how they can improve the accuracy of prediction in tropical maize hybrids. Our roadmap starts with the efforts for germplasm characterization, moving on to the practices for mating design, and the selection of the genotypes that are used to compose the training population in field phenotyping trials. Factors including population structure and the importance of non-additive effects (dominance and epistasis) controlling the desired trait are also outlined. Finally, we explain how the source of the molecular markers, environmental, and the modeling of genotype–environment interaction can affect the accuracy of GP. Results of 7 years of research in a public maize hybrid breeding program under tropical conditions are discussed, and with the great advances that have been made, we find that what is yet to come is exciting. The use of open-source software for the quality control of molecular markers, implementing GP, and envirotyping pipelines may reduce costs in an efficient computational manner. We conclude that exploring new models/tools using high-throughput phenotyping data along with large-scale envirotyping may bring more resolution and realism when predicting genotype performances. Despite the initial costs, mostly for genotyping, the GP platforms in combination with these other data sources can be a cost-effective approach for predicting the performance of maize hybrids for a large set of growing conditions.

## Introduction

Hybrid breeding programs are usually based on pureline methods, including the development of inbreeding lines by self-pollination or double-haploids, followed by progeny evaluation across heterotic pools (Hallauer et al., 2010). The great challenge of this approach is to adequately test the performance in all possible combinations of lines in crosses (Bernardo, 1994). In this context, we have conducted several studies indicating the usefulness of genomic prediction (GP, Meuwissen et al., 2001). Since the first studies of GP in maize (Bernard and Yu, 2007), several applications have been made to improve different steps of maize breeding, such as the selection under diverse breeding populations (Lorenzana and Bernardo, 2009; Lehermeier et al., 2014), the rapid cycle improvement of parental inbreeds (Zhang et al., 2017; Cui et al., 2020; Das et al., 2020), the prediction of double-haploid lines (e.g., Cooper et al., 2016; Messina et al., 2018), and the prediction of the performance of single-crosses for single or multi-environment conditions (Windhausen et al., 2012; Dias et al., 2018; Alves et al., 2019; Millet et al., 2019; Costa-Neto et al., 2020; Rogers et al., 2021).

Here we focused our review efforts on the GP of maize hybrids, particularly in the single-crosses of F_1_. From the last 10 years of research in this field, several research groups pointed to affect the main factors that drastically affect the accuracy of GP for hybrid prediction, such as (1) the genetic design and the genotypes used to form the training population; (2) the presence of a population structure; (3) the importance of non-additive effects controlling the desired characteristic; (4) the source of molecular markers used; and (5) the genotype × environment (G × E) interaction over contrasting environments. Therefore, this review aims to describe the most important outcomes in this field and report our research experience in a small-scale low budget breeding program under tropical growing conditions.

## Roadmap for Implementing GP in Hybrid Breeding Programs

Here, we highlighted the most important outcomes obtained by the Allogamous Breeding Laboratory of the University of São Paulo (USP, Brazil) and some other groups in testing GP for predicting maize hybrids. We present our review as a roadmap for small-scale and low-budget breeding programs due to the fact that most of our research is focused on optimizing GP in order to find the best training sets (TS), to select the best genotyping pipelines, and to choose the best multi-environment structures to predict scenarios of genotype × environment interaction. Our roadmap began with the efforts for germplasm characterization, which involves both molecular and phenotypic characterization. Before this step, it is necessary to develop the inbred lines during successive cycles of self-crossing. For most breeding programs, this step may involve the use of double haploid technology. After seed replication, field trials must be well-conducted, following certain management practices, which may evolve, for example, the use of optimum vs. nitrogen-limited conditions. A good statistical analysis and phenotype correction are important steps that impact further genomic analysis (Galli et al., 2018).

Then, after the characterization of lines, we focused on maize hybrid predictions. The second step of the roadmap considers schemes for mating design and choosing the genotypes used to compose the training population in field phenotyping trials. Factors including population structure and the importance of non-additive effects (dominance and epistasis) controlling the desired trait are also outlined. Finally, we present how the source of the molecular markers, environment, and the modeling of genotype × environment interaction can affect the accuracy of GP. We also point out that the use of dominance effects in GP is crucial to deliver accurate predictions of maize hybrids. Results of 7 years of research in our public maize hybrid breeding program under tropical conditions are discussed, and with the great advances that have been made, we find that what is yet to come is exciting. In the end, we revised some fields of work and the lessons we learned from both our experience and the results from other groups.

## Germplasm Characterization

### Tropical Germplasm of USP, Brazil

The very first step on our scientific road was to carry out germplasm characterization on the newly acquired inbred lines (Sant'Ana et al., 2020). Genomic diversity and population structure of germplasm (e.g., heterotic groups) are widely known to accelerate genetic gains in breeding programs. This structure and diversity are allocated to two major groups, such as temperate and tropical germplasm in tropical maize. While tropical maize germplasm has a greater genetic diversity, the temperate one has more pronounced heterotic patterns (Mir et al., 2013). Moreover, tropical maize germplasm lacks information on its genetic diversity regarding low-nitrogen (N) stress (De Andrade et al., 2016; Torres et al., 2018). In this context, in order to analyze the population structure of tropical maize accessions and identify genomic regions related to low-N tolerance, an initial set of 64 inbred lines was evaluated under ideal and low N availability conditions. The lines were genotyped using 417, 112 Single Nucleotide Polymorphism (SNP) markers from the Affymetrix platform described above. The grouping, based on the Nitrogen Acquisition Efficiency (NAE) values, classified the lines into two phenotypic groups, the first of which was composed of genotypes with high NAE (called H_NAE group) and the second of genotypes with low NAE (called L_NAE group). The groups H_NAE and L_NAE presented mean NAE values of 3,304 and 1,644, respectively (Sant'Ana et al., 2020). The population structure analysis revealed a weak relationship between genetic and phenotypic diversities. Simultaneously, line pairs having a high NAE and a considerable genetic distance were identified.

In greater detail, we noticed that a set of 29 single nucleotide polymorphism (SNP) markers displayed a significant difference in the allelic frequencies (Fst > 0.2) between groups H_NAE and L_NAE. Pearson's correlation between NAE and the favorable alleles in this set of SNPs was 0.69. These SNPs can be useful for the marker-assisted selection (MAS) for low-N tolerance in maize breeding programs. The results of this study can assist maize breeders when identifying genotypes to be used in the development of low-N tolerance cultivars.

Using this information, we have chosen 49 lines to compose the genitor bank of our breeding programs. We carried out the first complete diallel, which outlined the heterotic groups and the first GP training population, both used in the studies described below.

### Finding Population Structure in Hybrid Breeding Populations

**Figure 1:**
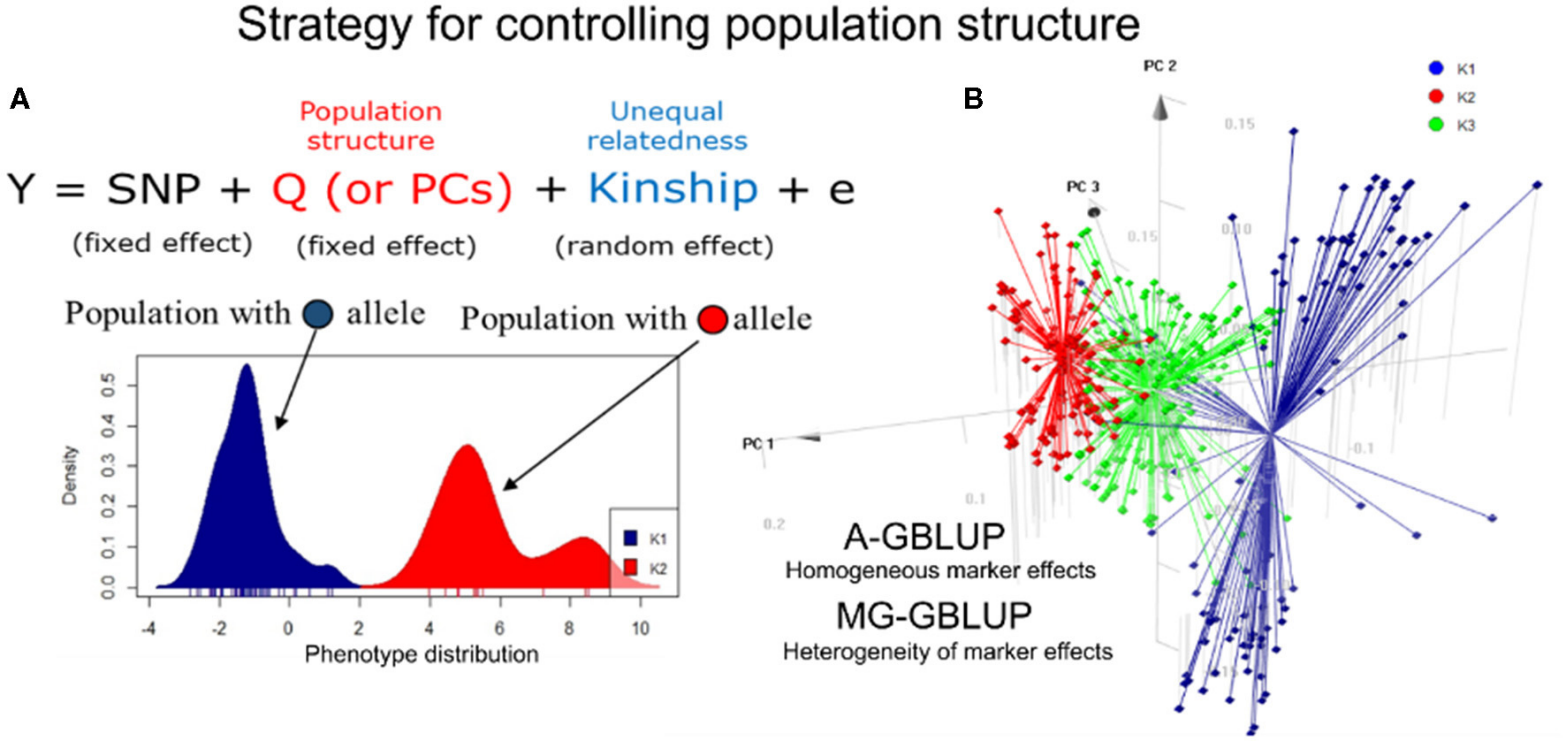
Approaches to control the maize population structure. **(A)** A mixed linear model accounts for the covariates of population structure (fixed effect) and the genomic relationship matrix (kinship). An example of an allele-specific the population is shown in the graph. **(B)** 3D graph for the first three major components (PCs) using 452 simple tropical maize hybrids. Two stratification methods for the prediction of hybrids are shown in the panel. The first is a homogeneous group approach (A-GBLUP), which assumes constant marker effects between groups. The second is a multivariate approach (MG-GBLUP) that uses data from several groups and considers heterogeneity, with population-specific marker effects that can be correlated between subpopulations.

Due to the considerable diversity, we then tried to identify whether the population structure within the dataset should be considered (Lyra et al., 2018). Population structure arises mainly due to geographical isolation and natural/artificial selections. Individuals are distributed into a few to several distinct subgroups that display different allele frequencies (Figure 1A). In a genome-wide association study (GWAS), individuals within the diversity panel present a specific phenotype of one or more lines that may generate misleading estimates on the linkage imbalance. As a result, whenever a phenotype is correlated with a subpopulation, this phenotype will probably show spurious associations. Although these associations are a major concern for GWAS, the use of highly structured subgroups in hybrid prediction could influence the achievement of reliable estimates of genomic estimated breeding values (GEBVs) for quantitative traits (Larièpe et al., 2017; Werner et al., 2020).

There are many ways to account for population structure in GP. Traditionally, the use of only a genomic relationship matrix is enough to predict phenotypes within breeding populations. However, when there is a strong structure (e.g., diverse panels), one strategy is to incorporate autovectors and admixture coefficients as covariates (fixed effects) in genomic models (Figure 1A). The use of principal components (PCs) in the genomic best linear unbiased prediction (GBLUP) method might result in a poorly positioned model because PCs enter both as fixed effects and implicitly, *via* the random effect (de Los Campos and Sorensen, 2014). Another option is to consider population structure in the cross-validation scheme, ensuring that each subpopulation is equally represented in the training and validation sets, consequently maximizing relatedness (Atanda et al., 2021). A third approach essentially divides the population into homogeneous (putative unstructured) subgroups (Figure 1B). When predictions are limited to specific subpopulations, the predictive ability is generally greater than predicting between subgroups or correcting for PS covariables (Guo et al., 2014). On the other hand, despite efforts to control the heterogeneity of marker effects among subpopulations (e.g., MG-GBLUP model, Lehermeier et al., 2015), dividing the population into subgroups may lead to a reduction in population size and a loss of diversity, thus reducing the predictive ability.

Tropical and subtropical maize genotypes are not as organized as temperate ones, which mean that more than two heterotic pools can be used in crosses. Equivalently, a diverse population of inbred lines can be crossed with testers representing different genetic origins. Thus, although only the effect of alleles and their interactions make up the genetic structures of hybrids, it is essential to find the structure patterns and understand how this information affects the predictions. In this sense, we investigated the effect of population structure in the GPs of simple crossbreeding considering two scenarios: (1) applying the traditional GBLUP and four methods of adjusting population structure in the whole group and (2) using homogeneous (A-GBLUP), within-group analysis (W-GBLUP), multi-group analysis (MG-GBLUP), and inter-group analysis (AC-GBLUP) in stratified groups (Lyra et al., 2018).

No advantages were found in the addition of population structure covariables to the prediction model based on the predictive ability. Thus, one explanation could be that the genomic relationship matrix has implicitly captured the genetic variation of population structure and hybrid mixing; another reason could be the similarity in the average performance of the characteristics in the subpopulation. Our second strategy was to divide the population into stratified groups. From our results, the predictive ability was significantly higher in A-GB and MG-GBLUP than W-GB for both characteristics, suggesting that considering the heterogeneity of the marker effects among subpopulations may be a promising strategy.

Our results suggest that the population structure problem for the GP can be efficient for highly structured (defined) populations but not for single hybrids. These results provided further knowledge about our germplasm and reassuring ways to perform GP.

## Design of Training Populations for Genomic Prediction

### Finding the Best Mating Design for Training Populations

*Post-hoc* but relevant information about creating a training population is included in our realm of projects. We realized that the literature concerning GP in maize was quite vast, yet there was a significant shortage of studies on the best genetic design to build the training population.

Therefore, we handled a study to verify genomic selection accuracy to predict the performance of maize hybrids under different genetic designs (Fristche-Neto et al., 2018). Several mating designs, such as Griffing's methods, partial diallel, North Carolina Design II (NCII), and test crossing (Hallauer et al., 2010) have been proposed. These methods have the following four main goals: (i) to provide information on the genetic control of the trait under investigation; (ii) to generate populations to be used as a basis for the selection and development of cultivars; (iii) to provide estimates of genetic gain; (iv) to obtain information to evaluate the genitors used in the breeding program, based on the general and combination-specific capabilities (GCA and SCA), respectively. Although many articles have been published on GP in maize (Lorenzana and Bernardo, 2009; Windhausen et al., 2012; Lehermeier et al., 2014; Cooper et al., 2016; Zhang et al., 2017; Dias et al., 2018; Messina et al., 2018; Alves et al., 2019; Millet et al., 2019; Costa-Neto et al., 2020; Cui et al., 2020; Das et al., 2020; Wang et al., 2020; Rogers et al., 2021), no studies on the best genetic design to build the training population have yet been conducted. This population should maximize the accuracy and contemplate practical restrictions, such as the costs and logistics of crosses to be made. Thus, in this study, we aimed (i) to empirically evaluate the effect of genetic designs when used as a GP training population of single maize hybrids obtained through full diallel (FD) or *via* NCII, and (ii) to identify the possibility of reducing the number of crosses and genitors to compose these TSs (Fristche-Neto et al., 2018).

In addition to the standard genetic designs, we also evaluated the possibility of using optimized training populations (OTS) aiming to reduce the number of individuals for training genomic prediction without reducing accuracy. For this purpose, we used the algorithm proposed by Akdemir et al. (2015) with predefined population size. Therefore, to predict the FD, we used the NCII, the testcross (TC), and OTS as the TS with sizes of 32 (with the same size of the TC data set), 152, 272, and 393 hybrids (with the same size as the NCII data set). Following the same idea of aiming to predict NCII, we used the TC and OTS with 32, 152, and 272 hybrids.

Our results suggest that TC is the worst genetic design to be used as a TS to predict simple maize crosses that must be obtained through FD or NCII. On the other hand, NCII is the best TS for the prediction of hybrids taken from FD. In addition, combinations from FD or NCII can be well predicted using OTS, thus reducing the total number of crosses to be made. However, the number of parents and crosses per parent in the ST should be maximized.

### Training Populations Using Public Databases—An Alternative

Due to the scarcity of resources in the initial phases, we addressed the possibility of incorporating public databases in the composition of our training populations (Morais et al., 2020). Small-scale public and private programs with limited budgets often lack the financial ability to genotyping a considerable number of individuals to apply GP efficiently. In this regard, Morais et al. (2020) have evaluated the usefulness of incorporating public database panels to compose tropical GP training populations. In this context, the following public databases were used: (a) ASSO—Nested Association Mapping Population (NAM) combined with the Maize Association Panel 282 (166 + 282 endogamic lines, respectively); (b) NCRPIS—United States Department of Agriculture—Agricultural Research Service (USDA-ARS), North Center Regional Plant Introduction Station (2.046 endogamic lines); (c) USP—tropical endogamic lines of the University of São Paulo (64 endogamic lines).

These databases contained phenotypic information regarding plant height (PH, in cm), ear height (EH, in cm), and the SNP markers data. A total of 29 training populations (TPs) were defined and divided into four scenarios to determine the best strategy to apply public databases to predict lines.

The best predictions were achieved with the strategy of the TP composed by candidates selected with an optimization algorithm from all the public database and private lines, even at the smallest TP sizes evaluated (81 and 281 TP sizes). On the other hand, the lowest predictive abilities were achieved using only the Tropical USP database as training and validation populations (VP), due to its lack of genetic variability and reduced population size, hindering prediction. The results of all four scenarios of TP formation showed that the predictive ability increased with the increase of TP size, the relationship rate between TP and VP, and genetic variability. (Rife et al., 2018) revealed a similar potential of GP to predict wheat traits using historical data across several public breeding programs, reinforcing the possibility of using external data for model training.

The optimization of the training population proposed by Akdemir et al. (2015) showed promising results, even when the training population size was reduced. For example, small groups of individuals (250) selected in public panels are enough to achieve predictive abilities of over *r* = 0.44 and *r* =0.53, for PH and EH, respectively. Optimizing the TP can increase the representation of the subpopulation, allowing for an efficient and controlled updating of the training population over the years (Akdemir et al., 2015).

Nevertheless, what is the real reason to use public databases, and how does it fit into a breeding framework? The use of public data aims to an early-start GP with reduced costs and over the years, to setup a more complex GP training population. The number of individuals from the program genotyped and phenotyped will increase as time goes on, reducing the participation of public databases in the training population and thus paying off the costs of genotyping the population in training over the years. For example, the total cost of the training population could be divided over 5 years, with the public database replacing 20% per year of the training population with individuals from the program.

Considering a training population that is 10 times bigger than the VP, this strategy should be conducted as follows: in the first year, (a) genotyping and phenotyping of the germplasm program, composing 10% of TP, along with external individuals selected by optimization procedures (90% of TP), (b) out of 10%, established as the VP (new progeny with no phenotyping data), (c) validation and prediction of GEBV. In the second year, (a) genotyping and phenotyping of individuals from the germplasm program (10% of the TP), (b) once again, new individuals are to make up the VP (10% of TP), while the remaining individuals from the germplasm program are to be a part of TP, with the TP composed by 70% of external individuals selected from optimization procedures and 30% of internal individuals genotyped previously, (c) validation and prediction of GEBV. As genotyping will be performed annually, after 6 years, the TP would be composed exclusively of individuals from the program. In the sixth year, the best performer could optimize the training population with internal individuals, maintaining a good prediction ability index. This procedure optimizes the technical, operational, and financial balance, considering the resources available over time and each harvest.

## Searching for New Sources of Markers and Reference Genomes

### Impact of the Genotyping Platform in GP

Nowadays, SNPs are the most widely used molecular markers in genomic studies, as they are abundant and evenly distributed in the genome. In addition, genotyping platforms that provide many markers have quickly, accurately, and cost-effectively allowed for the use of molecular tools, including GP. High-performance genotyping platforms, such as SNP-array and next-generation sequencing (NGS) provide thousands of markers for hundreds of samples, making them very suitable (Rasheed et al., 2017) for this purpose. Since there are different technologies to be detected, SNP-type markers can be different and located in distinct points of the genome so that later genomic studies can be affected by them. Recent studies have suggested comparable GWAS results, genetic diversity, and GP using different genotyping platforms in several species, including maize (Elbasyoni et al., 2018; Darrier et al., 2019; Negro et al., 2019; Chu et al., 2020) (Table 1).

**Table 1:** Reports on the comparison between GBS and array regarding genomic studies.

**Compared platforms**	**Species**	**Method**	**Overall result**	**References**
GBS and array	Wheat	GP	GBS comparable to or better than an array	Elbasyoni et al., 2018
GBS and array	Barley	GWAS	Broadly similar conclusions	Darrier et al., 2019
SSR, GBS, and array	Wheat	GP and diversity	Array underestimates diversity measures; similar predictive abilities	Chu et al., 2020
GBS and array	Maize	GWAS	Platforms were complementary for detecting QTL	Negro et al., 2019
GBS and array	Maize	GP	Similar results depending on the prediction model	Sabadin and Fritsche-Neto, 2020

*QTL, quantitative trait loci*.

In this context, we studied how SNP markers obtained from two genotyping platforms (616K SNP-array and GBS) affect the GP in our germplasm (Sabadin, 2020). We also attempted to verify the effect of the use of different reference genomes in SNP calls *via* GBS (i) using the most common reference genome, line B73 (GBS-B73), (ii) using a simulated reference genome built with GBS data, considering all inbred lines (GBS-Mock-All), and (iii) using a simulated reference genome built with GBS data from a single line, our heterotic pool tester L56 (GBS-Mock-L56). For this purpose, we used the USP data set mentioned above (see section above “Training populations using public databases”). To build the simulated genome, we used a pipeline developed by Melo et al. (2016), which captures the polymorphism regardless of an external genome. Finally, for each set of SNP marker data obtained from different platforms and approaches, we performed the GPs considering both the additive (GBLUP additive) and the additive-dominance (GBLUP additive-dominance) models.

### Density and Distribution of SNPs

The density and distribution of SNP markers varied according to the genotyping platform chosen. In our study, the SNP markers discovered by SNP-array and GBS-B73 had the same reference genome, which allowed us to compare them regarding marker distribution on chromosomes and detect coincident SNP as well. Despite the difference in the number of SNP markers (62,409 for SNP-array and 5,594 for GBS-B73), both platforms had similar distributions along the genome. However, only 300 SNP markers coincided, suggesting that they detected polymorphisms in different regions. Although this is an important result, these differences were only consistent for some GP models.

The GBLUP model is based on the genomic relationship between genotypes to estimate the genetic values of non-phenotyped individuals. Therefore, assessing the genomic relationship is more important than the polymorphism resolution, which was confirmed when we evaluated the additive genomic relationship matrix (Ga) and the genomic dominance matrix (Gd). For the Ga matrices, high correlations were observed between the SNP-array, GBS-B73, and GBS-Mock-All SNP data sets (*r* = 0.88), revealing that these approaches estimate the additive genomic relationship between hybrids in a similar way. However, for the Gd matrices, lower correlations were observed among all SNP data sets, which show that the polymorphism captured by these platforms estimated the dominance effects differently. GBS-Mock-L56 displayed low correlations with other SNP data sets and had a low performance for all downstream analyses, proving that it is an erroneous alternative to sample polymorphism within the population, since only polymorphisms between L56 and other individuals were identified. This information is crucial when the aim is to predict the genetic values of hybrids, although the architecture of the feature can influence the performance of GP models.

**Figure 2:**
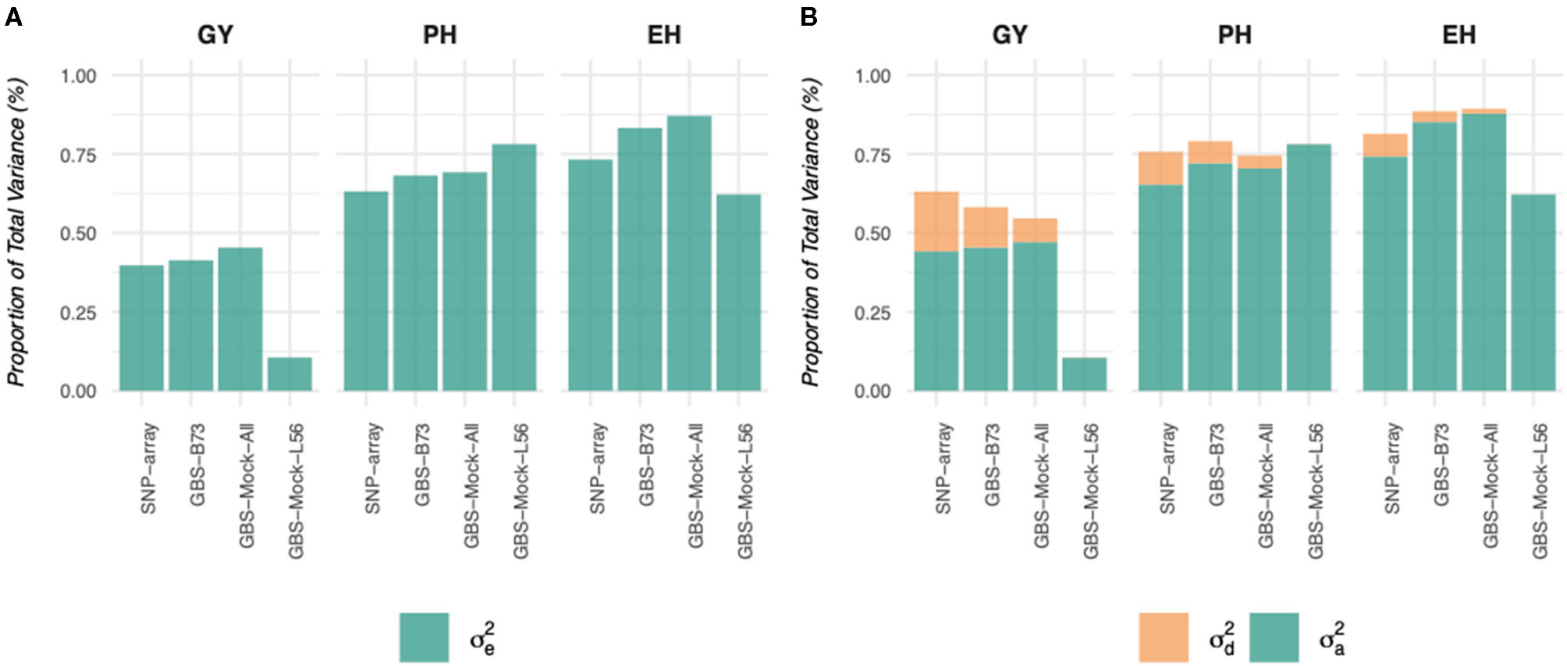
Proportion of the phenotypic variance explained by the estimated components of variance in the different traits (EH, ear height; PH, plant height; GY, grain yield), models and scenarios studied.

Similarly, when considering the variance captured by the additive effects and the dominance deviations, these proportions also vary depending on the genotyping platform and the genetic architecture of the characteristic (Figure 2), which can be explained by the reduction in the number of markers, which consequently inflates the effective size of these markers. On the other hand, the SNP-array captured higher proportions of total variance and dominance, yet it was close to zero in the GBS-Mock-L56, considering all characteristics. In addition, the differences for grain yield (GY) were more significant than for simple characteristics (plant and ear heights).

**Figure 3:**
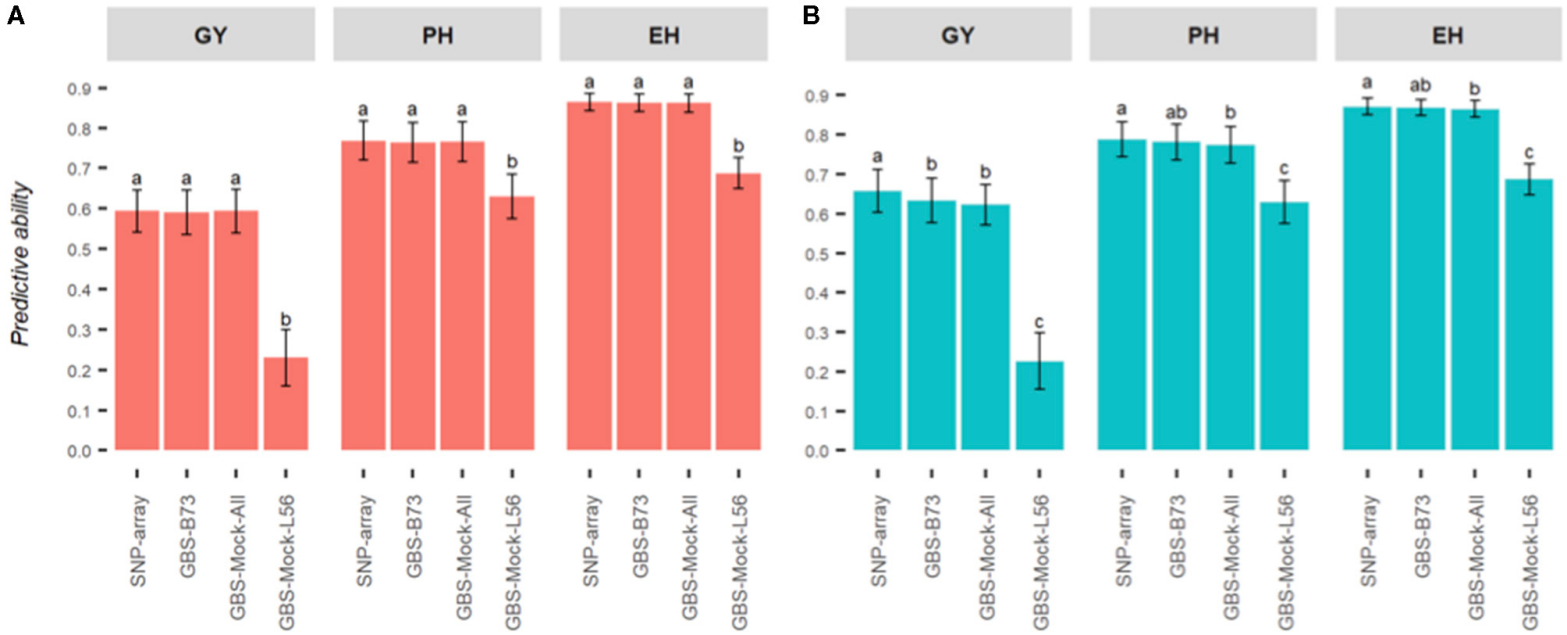
Summary of the predictive abilities for each combination of model and genotyping scheme studied for three agronomic traits in maize (EH, ear height; PH, plant height; GY, grain yield).

As far as predictive abilities are concerned (Figure 3), genotyping platforms and reference genomes do not affect the additive model, except for GBS-Mock-L56. Furthermore, the use of a reference genome historically unrelated to the evaluated germplasm, such as the B73 genome (temperate maize), seems to be enough to capture the additive relationship of the genotypes within the population.

This situation can change greatly when we consider the effects of dominance to estimate genetic values. In our study, except for GBS-Mock-L56, small differences in predictive capabilities were observed among SNP data sets, when we performed the GBLUP additive-dominance model. Furthermore, the differences were more remarkable for GY, supporting the fact that the inclusion of the dominance effects of GP models is more relevant for complex traits. The coefficients of determination between GEBV estimates remained high (the lowest was for GY, *R*^2^ = 0.88) but below that when obtained with the additive model.

Finally, for GP purposes, the most common genotyping platforms (SNP-array and GBS) offer very similar predictive abilities when using only additive effects in GP models. However, when we add dominance effects, their performance may change, especially when estimating hybrid performance. Dominance effects are critical to hybrid GP, and therefore, the choice of a genotyping platform may affect the estimates of genetic values. However, the differences appear to be small and acceptable in some cases. Furthermore, the use of a reference genome historically unrelated to the evaluated germplasm does not seem to be a decisive factor for GP since it can sample the haplotype variability among genotypes within the population. Another highlight uses a simulated reference genome to discover SNP since it does not depend on an external genome to detect polymorphisms. This strategy may be a valid alternative when conducting GP studies with reliable estimates, especially for orphan crops, where a reference genome is not yet available. Somehow, sampling polymorphisms consistently, using all genotypes within the population, is recommended to build the simulated genome.

## Genetic Architecture and Further Genomic Prediction Modeling

### Connecting Phenotypic and Genomic Variation

Once optimal germplasm characterization, population structure, training population mating design and composition, and genotyping methodology were defined, there was interest in further improving predictive abilities through modeling (Alves et al., 2019, Galli et al., 2020). The ability of the GP to connect phenotype and genotype has been proven to have a strong relationship with the genetic architecture of the trait. In this sense, tools such as GWAS have been applied, and the results have suggested the existence of a wide range of genetic control patterns in agronomic traits. Thus, many GP methods have been proposed to address the domain of genetic architectures. However, for open pollination species, such as maize, while the identification of variants and architectures by GWAS is usually performed in inbred lines, the GP is mainly directed at selecting hybrids. In this sense, the usefulness of *a priori* GWAS in lines to predict its hybrid offspring has been explored by Galli et al. (2020). The trait used in the case study was the low-nitrogen tolerance index (LNTI).

**Figure 4:**
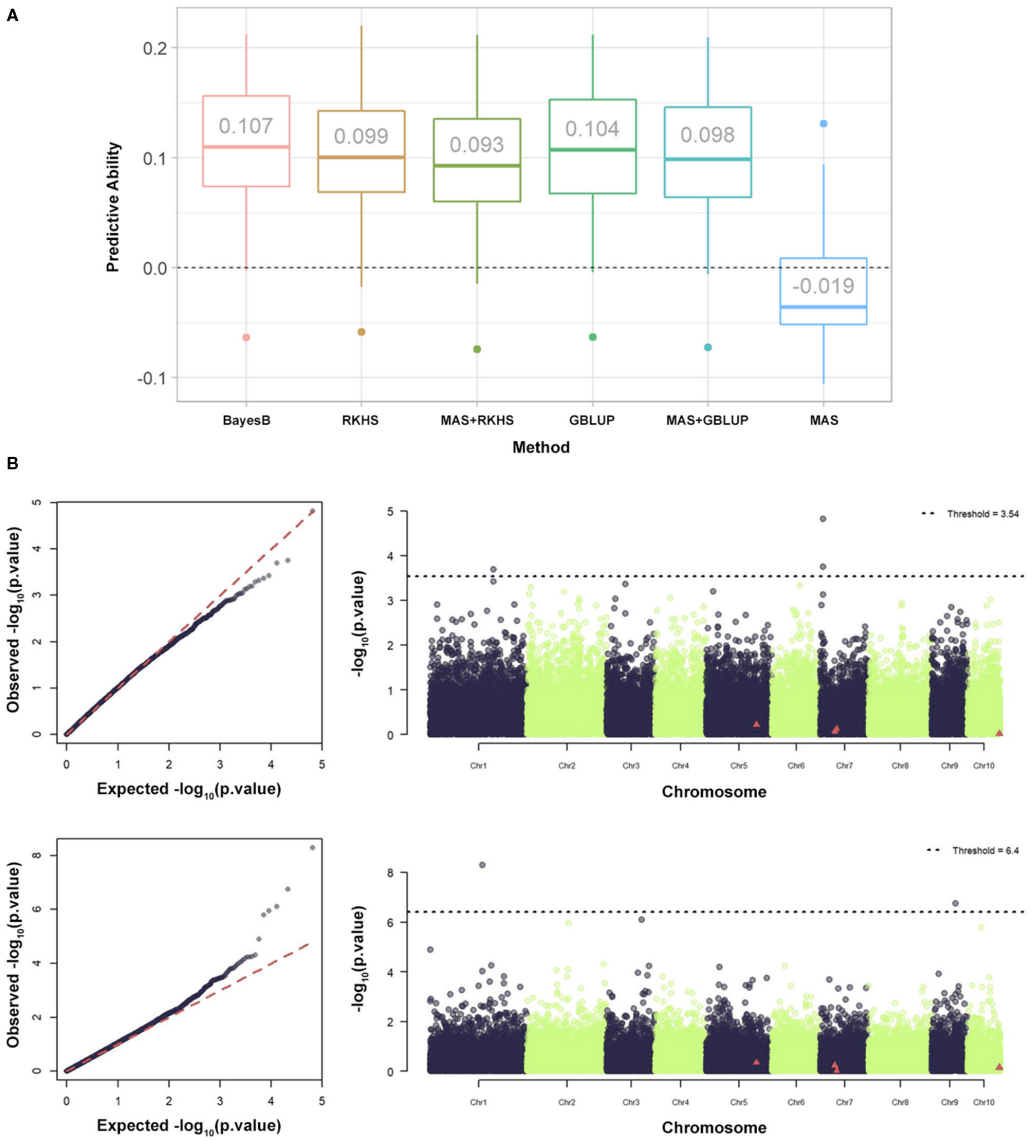
Performance of different statistical models and GWAS-based strategies for genomic prediction of maize hybrids. **(A)** Summary of the predictive capabilities of the Low Nitrogen Tolerance Index (LNTI) in maize hybrids using BayesB, RKHS, MAS + RKHS, GBLUP, MAS + GBLUP, and MAS additive. **(B)** Summary of GWAS, QQ, and Manhattan graphs for LNTI. The graphs represent additive GWAS (upper) and dominance (lower). The MAS was based on statistically significant associations identified for LNTI by Morosini et al. (2017).

In previous GWAS (Morosini et al., 2017), four significant trait marker associations were identified in the parental population. The influence of these associations was verified for MAS, GP, and the MAS + GP of hybrids (Figure 4). The GP was performed with all molecular markers, except when associated with the MAS. For MAS + GP, the significant markers were removed before calculating the genomic relationship matrices. Three GP methods, namely BayesB, GBLUP, and RKHS (Figure 4A). Finally, GWAS was performed considering the additive, dominance, and additive and dominance in hybrids to verify the coincidence of associations with the parental lines (Figure 4B). The predictive ability of LNTI was observed to be low, ranging from −0.019 to 0.107 (Figure 4A). It was also shown that (i) the MAS of hybrids with markers identified in inbred lines had the lowest predictive abilities; (ii) adding *a priori* information from inbred lines of GWAS decreased the predictive ability of GP (MAS + GP); (iii) GP alone produced the best results.

To date, many studies have found that GP accuracy can be enhanced using *a priori* information, especially from GWAS (Zhang et al., 2014; Spindel et al., 2016). However, the results are conditioned by factors, such as trait heritability and the variation explained by the main genes (Bernardo, 2014). Furthermore, the results obtained by Galli et al. (2020) corroborate the long-standing hypothesis of the lack of connection between inbred lines and the performance of their hybrid offspring. In addition, the GWAS of hybrids produced different marker-trait associations to those found for the parental lines published in 2017. The differences observed were both the nature of intralocus interaction and the location of markers, suggesting that the most important genes driving phenotypes in inbred lines and hybrids might be different.

### Understanding the Impact of Heterosis in GP

According to Sprague and Tatum (1942), hybrid performance can be divided into two components, namely general combining ability (GCA) and specific combining ability (SCA). The GCA component can be explained by the differences between the average performance of parental lines in crosses and the average of the overall population. In this sense, the GCA of a line depends on the substitution effects of the allele and involves additive and non-additive genetic effects (Reif et al., 2007). The SCA, on the other hand, represents the deviation of hybrid performance from parental averages. This component is often attributable to deviations from additivity due to dominance and epistasis (Reif et al., 2007), and it is one of the most critical components of hybrid performance. Thus, the additive and non-additive effects of markers must be estimated to consider all the genetic variance present in a population.

The modeling of non-additive effects in genomic studies can provide several advantages (Technow et al., 2012; Varona et al., 2018), such as (1) increasing the accuracy of prediction of genomic selection methods, (2) allowing for the allocation of crossover and consequently, and (3) a better exploration of heterosis (Kadam et al., 2016). However, one of the barriers is that additive and non-additive effects are often not mutually orthogonal. For this reason, the parameters of variance that enter genomic models (for example, the additive and the dominance variances) cannot be used directly to break down total genetic variance into GCA and SCA components. As presented by Alves et al. (2019), due to their flexibility, Bayesian models can be used to estimate these important parameters, especially when the genetic design does not allow an orthogonal decomposition of genetic variance in these components.

In this context, Alves et al. (2019) presented a method to decompose genetic variance into GCA and SCA using Bayesian genomic models that account for additive and non-additive effects (dominance and epistasis).

**Figure 5:**
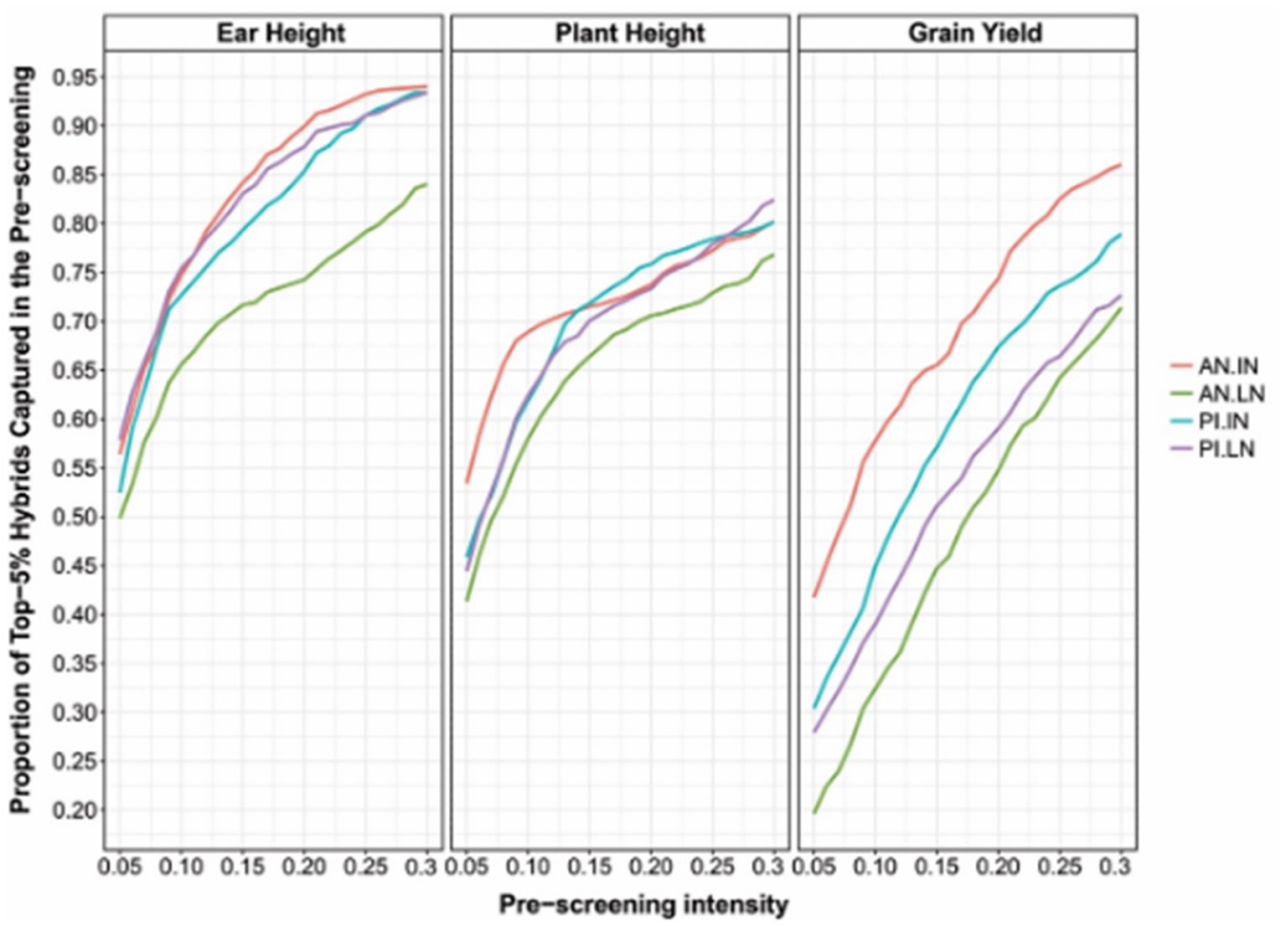
According to phenotypic classification, the proportion of 5% higher hybrids was identified by pre-screening based on cross-validation via GP using the additive + dominance model at a certain selection intensity (x-axis). Each panel corresponds to one evaluated character. The lines within a graph represent different environments (AN: Anhembi; PI: Piracicaba; LN: Low nitrogen; IN: Ideal nitrogen).

The proposed method can be applied not only to single hybrids but also to double and triple hybrids. As proof of concept, the proposed approach was applied to the data set described above (USP, see section Germplasm Characterization). The results showed that non-additive effects play a crucial role in expressing quantitative characters under stress conditions (especially GY, Figure 5). This study also showed that the accuracy of the prediction models that account for the additive and non-additive effects depends on interest characteristics. It was also found that selecting 30% of the best single-crosses during the pre-selection phase in the field, based on GP with additive and non-additive effects, leads to a subset of hybrids that contained 85–95, 70–80, and 75–85 of the 5% higher hybrids for ear height, plant height, and GY (Figure 5), respectively.

## Modeling Genotype × Environment Interaction (G × E) in GP

### Finding Novel Kernel Methods and Modeling Structures for G × E

The G × E is a multiplicative non-additive effect due to the non-parallel trait-specific phenotypic responses, a function of genotype diversity and environmental variation. Since 2012, when the marker by environment interaction approach was developed (Burgueño et al., 2012), the analysis and modeling of G × E have evolved from the genotype to the gene or genomic level (Crossa, 2012). However, multi-environment modeling to predict maize hybrids started with Dias et al. (2018) (Table 2). Since then, several efforts have been made to extend those modeling approaches when considering different kernel methods and structures. For example, different G × E approaches to include genomics and large-scale environmental data (enviromics) (Bandeira e Sousa et al., 2017; Costa-Neto et al., 2020; Rogers et al., 2021) using explicit covariates for modeling reaction-norms (Millet et al., 2019) or implicit covariates derived from multivariate structures (e.g., Dias et al., 2018; Krause et al., 2020).

**Table 2:** Strategies and main results for multi-environment genomic prediction of grain yield, the main agronomic trait in hybrid maize breeding since 2017.

**Germplasm**	**Core ideas and importance**	**References**
Tropical hybrids	The first use of GP for modeling G × E and predicting maize hybrids	Acosta-Pech et al., 2017
	Differences of several variance–covariance structures and Gaussian kernel in the prediction of G × E	Bandeira e Sousa et al., 2017
	Contribution of dominance effects and factor analytic structures for G × E	Alves et al., 2019
Temperate DH lines	The use of crop models with genomic prediction (CGM-WGP) is better than GBLUP	Cooper et al., 2016
	Update of CGM-WGP and application in predicting phenotypic landscapes	Messina et al., 2018
Temperate hybrids	Use of factorial regression to find covariates that explain genomic-enabled reaction norms	Millet et al., 2019
Tropical hybrids	Deep kernels accounting for genomic and near-infrared relatedness kernels	Cuevas et al., 2019
	The importance of additive (A), dominance (D), and AA, DD, and AD covariances under Bayesian prediction approaches	Alves et al., 2019
	The use of deep kernel and Gaussian kernel for modeling additive and dominance G × E effects with reaction norm	Costa-Neto et al., 2020
	Multivariate GBLUP using factor analytic structures	Krause et al., 2020
Temperate hybrids	The use of dominance and functional enrichments to increase GP	Ramstein et al., 2020
	The use of difference variance–covariance structures to model dominance and reaction-norm	Rogers et al., 2021
Tropical hybrids	Contribution of non-additive effects and mega-environment grouping in prediction accuracy	Alves et al., 2021

Our research group aimed to understand how environmental characterization (envirotyping) and non-linear kernels could improve prediction models, including G × E (Bandeira e Sousa et al., 2017; Costa-Neto et al., 2020). Below, we detail a case study using our tropical maize germplasm from USP, in which we were able to test novel G × E structures and kernel methods to model genomic × environment effects.

We conducted an extensive study on G × E over three agronomic traits in tropical maize (GY, PH, and EH) for two different sets in Brazil. Bandeira e Sousa et al. (2017) tested two kernel methods, a linear (GBLUP, hereafter abbreviated as GB) and non-linear (Gaussian Kernel, GK) kernel and four modeling structures for G × E using (i) single-environment (SE) model, using the average values of the genotypes for all environments; (ii) multi-environment, main genotypic effects model (MM); (iii) multi-environment, single variance G × E deviation model (MDs), and (iv) multi-environment, environment-specific variance G × E deviation model (MDe). Models without G × E structures (SM and MM) were less accurate than those including G × E effects (MDs and MDe). For the MM, MDs, and MDe models, the increase in the prediction accuracy of GK over GB ranged from 9 to 49%. As expected, GY was the less predictable trait due to its polygenic nature, and because of that, this trait became the main target for further studies. For all traits, few differences were observed between the MDs and MDe models. Gaussian Kernel was observed to outperform all GB-based models in accuracy for all models, with an average accuracy gain from 34 to 70%. However, for EH and PH, the gains using GK were smaller than using GB.

### Understanding the Contribution of Non-additive Effects for G × E

Since 2017, some studies have pointed that the use of additive (A) plus non-additive effects (e.g., dominance, D; epistasis, A × A) might drastically improve the accuracy of GP for maize hybrids (Acosta-Pech et al., 2017; Dias et al., 2018; Alves et al., 2019, 2021; Costa-Neto et al., 2020; Ferrão et al., 2020; Ramstein et al., 2020; Rogers et al., 2021), especially with G × E under multi-environment conditions. It seems that the main dominance effect (D) plus dominance by the environment interaction (D × E) corresponds to about 50% of the observed phenotypic variation for complex traits, such as GY in hybrid maize. This is an important issue because the usage or non-usage of non-additive effects only depends on the computational effort expected, that is, from raw molecular marker data, it is feasible and easy, nowadays, to compute both additive or non-additive effects and their relatedness-based matrices to implement GBLUP and kernel models (Alves et al., 2019). The use of algebra resources to remove the complexity of the variance–covariance matrices, such as the singular decomposition value (Costa-Neto et al., 2020; Cuevas et al., 2020) and factor analytic structuration (Dias et al., 2018; Rogers et al., 2021) is a computationally smart way to translate model complexity into accuracy gains. Here, we detail the results we found as an extension of the study of Bandeira e Sousa et al. (2017), related to the first option resource previously mentioned.

We investigated different models involving additive (A) and additive-dominance (AD) main effects (MM model, but using A + D), along with the interactions (MDs models) including reaction-norm for A and D effects to predict GY (Costa-Neto et al., 2020). After the use of GB and GK, a third kernel method was also tested, the so-called deep kernel (DK), which takes advantage of the arcsine kernel that thought the available phenotypic data could mimic different hidden layers an in-depth learning approach. Thus, DK is also a non-linear kernel, but unlike GK, it approaches the genomic relatedness into an empirical relatedness of the individuals across a diverse set of environments. Our results suggest that DK outperforms GB and GK when exploring dominance effects in hybrid prediction. In terms of explaining the phenotypic variation across multi-environment, the DK and GK models better captured the genomic and enviromic sources and reduced the residual variance of the models. Then, we tested three scenarios, namely CV1, novel genotypes in known environments; CV2, sparse MET conditions, some genotypes at some environments, and CV0, novel environments.

In addition, our results indicated that GK and DK explore the G × E variation better (in this case, G × E = A × E + D × E) in a less computationally expensive way than GB. The GB kernel was the worst kernel method for exploring D effects to predict GY in maize hybrids. For all prediction scenarios (CV1, CV2, and CV0), we observed that accuracy gains could only be achieved for GB-based models when including some envirotyping data as the main effect (W) or as reaction-norm (G × W = A × W + D × W). The non-linear kernels were also more efficient at using the phenotypic records in training models for CV1, CV2, and mostly for CV0. For CV0, the combination of DK and more straightforward reaction-norm models (including only A + D + W effects) achieved almost the same accuracy as more complex structures (A + D + W + A × W + D × W). This suggests that to predict future scenarios using actual TSs, the use of enviromic sources combined with additive and dominance genomic data, both modeled with non-linear kernels, is the best way to achieve higher mathematical accuracy biologically that better represents novel G × E conditions.

### Finding Novel Enviromic Approaches to Deal With G × E

Combined with phenotypic and genotypic data, the use of envirotypic data sources can leverage the molecular breeding strategies addressing the prediction of tested and untested environments, such as climate change scenarios (Millet et al., 2016, 2019; Messina et al., 2018; Bustos-Korts et al., 2019; de los Campos et al., 2020; Guo et al., 2020). These data have been incorporated into GP in the last ten years to better model the G × E interaction according to the reaction norm (Heslot et al., 2014; Jarquín et al., 2014; Gillberg et al., 2019; Costa-Neto et al., 2020; Rogers et al., 2021). However, it is difficult for most breeders to deal with this interaction between environmental models, ecophysiology, and genetics (Costa-Neto et al., 2021), in which we need to (i) implement a cost-effective and intuitive pipeline to integrate envirotyping data in GP and (ii) find novel enviromic approaches, more capable of describing phenotype-envirotype covariances and translate it into accuracy gains. Below, we briefly present the results by Costa-Neto et al. (2021), who implemented an envirotyping pipeline and then review some of the main applications of enviromic data achieved for other groups.

Costa-Neto et al. (2021) presented two novel approaches to modeling the environmental similarity from enviromic data. Using a proof-of-concept data set, we tested the importance of (i) EC-specific kernels for main environmental factors and (ii) the envirotyping level at each key development stage of crop development. For the latter, we proved accuracy gains of the reaction-norm models using a specific environmental relatedness, built using ECs for each development stage, concerning the benchmark environmental relatedness (single-environmental kernel using all ECs at all development stages). This approach enabled a better understanding of which development stage impacts the relatedness of individuals across MET. We tested a CV1 scheme to predict GY using a drastically reduced phenotyping level (only 20% of the phenotypes were used as TS). We showed that a model without enviromic data has a minimal prediction accuracy (*r* = 0.101), and the inclusion of envirotyping data boosted the prediction up to *r* = 0.504 (enviromic by development stage) and *r* = 0.485 (enviromic for all crop development stages).

An alternative approach for the use of environmental relatedness kernels is the adoption of single-covariate regressions (Ly et al., 2018) or the first step of screening in which the ECs that best explain the trait variation are used to fit a simpler but more accurate linear reaction-norm structure (Millet et al., 2019). These ECs can be collected from in-field sensors or public databases (for more details, see the next section) and also consider stress-covariates derived from crop growth models (CGM) (Heslot et al., 2014; Rincent et al., 2017). For the latter, a more robust single-step approach relies on the integrated use of GP with CGM, which was successful in predicting the performance of DH maize lines on water-stressed environments (Cooper et al., 2016) and across a large target region of the breeding program in the United States (Messina et al., 2018). For low-budget breeding programs that are unable to invest in large phenotyping for ecophysiology traits (e.g., biomass accumulation during crop life) need to improve accuracy in training CGM. An alternative can be in the exploring of the environmental relatedness or EC-specific regressions, which increases the accuracy of GP in hybrid prediction more simply (Costa-Neto et al., 2020; Rogers et al., 2021) with a satisfactory ability to predict cultivar responses (de los Campos et al., 2020) and explain the reaction-norm for both complex quantitative traits (Ly et al., 2018; Millet et al., 2019) and less complex traits (Guo et al., 2020; Jarquin et al., 2020).

## Open-Source R Packages to Facilitate the Adoption of Genomic Prediction

Since the first work on GP, published approximately 20 years ago (Meuwissen et al., 2001), a wide number of computational solutions have been developed to process data and run prediction models, such as *BGLR* (Pérez and de los Campos, 2014), *rrBLUP* (Endelman, 2011), and *sommer* (Covarrubias-Pazaran, 2016). For plant breeding, most of these solutions were implemented in R, an open statistical-computational environment. Nowadays, these software solutions can offer the processing of genotyping data (Granato et al., 2018b), fit marker regressions or genomic wide association analysis (Endelman, 2011), run GP accounting for several multi-trait multi-environment approaches (Pérez and de los Campos, 2014; Covarrubias-Pazaran, 2016; de los Campos and Gr?neberg, 2016; Granato et al., 2018a; Montesinos-López et al., 2019), and integrate envirotyping sources in the reaction-norm modeling of G × E (Costa-Neto et al., 2021). Here, we briefly discuss three software developed by the Allogamous Plant Breeding Laboratory of the University of São Paulo as part of our experience in the field of genomic-enabled prediction of maize hybrids.

To deal with genotyping data, we developed the package *snpReady* (Granato et al., 2018b), which helps the user with quality control and the recoding of markers. In addition, it helps obtain some parameters of population genomics. This package implements a pipeline of conversion, imputation of missing data, and preparation of genotyping data for genomic analysis, outputting matrices in appropriate formats for different software. These applications are simple and enough to be integrated into the breeding pipelines or coupled with other environments, such as shiny (Matias et al., 2019).

After that, we realized the need to implement a computationally efficient approach that facilitates the use of multi-environment prediction structures accounting for G × E. To fill this gap, we developed the package, Bayesian Genotype plus Genotype Environment (BGGE, Granato et al., 2018a), which considers a wide number of genomic environmental structures and two kernel methods (linear GBLUP and non-linear Gaussian kernel) in a processing time of five times faster than Bayesian Generalized Linear Regressions *(BGLR)*. Furthermore, it uses algebra resources resulting in a significant gain in processing speed, especially for large data sets (Granato et al., 2018a), such as near-infrared data (Cuevas et al., 2019), historical yield trial data (Cuevas et al., 2020), and enviromics (Costa-Neto et al., 2020).

For the latter, since the first work involving the use of environmental information in GP (Heslot et al., 2014; Jarquín et al., 2014), there is a need to fine-tune the methodologies of collection, processing, and the use of this data in GP. Generally, the collection, organization, and processing of environmental data are steps that require the installation of equipment in the field. In turn, such equipment may be expensive or difficult to access for some research groups in specific regions or countries. Therefore, we have decided to enter a routine of climate data collection through NASA's Prediction of Worldwide Energy Resources (NASA-POWER, Sparks, 2018), which can access information daily, anywhere in the world. Thus, the computational development of these routines evolved to the development of the first open-source envirotyping pipeline, named *EnvRtype* (Costa-Neto et al., 2021). Three modules of envirotyping are offered in this package, namely (i) the collection of raw environmental information from public platforms, requiring only the geographic and temporal coordinates of the experiments and processing data set, (ii) environmental characterization based on the use of the processed environmental covariables to describe the typology of the environments, and (iii) the implementation of GP models enriched with ecophysiological parameters, considering three different structures of reaction-norm, and subsequently incorporate them into the prediction models under a Bayesian framework in the same way as in *BGGE*.

## Final Remarks

This work aimed to present a review of our results, which shows that it is possible to increase the accuracy in the prediction of hybrids. This requires the use of optimized training populations, the inclusion of non-additive genetic effects in the prediction models, and environmental information to compose the matrices of G × E covariance and non-linear kernels of genomic relationship. On the other hand, there are no significant gains in the accuracy using GWAS information in parental lines, population structure, or using markers from new generation sequencing. Below, we conclude our work by describing some lessons we learned, both from our studies and other groups.

### GWAS Might Be Useful to Discover the Architecture of G × E for Further GP Modeling

Going back to our experience with GWAS described in this review, we found in our road map that the use of GWAS for further prediction modeling might be more successful, especially to understand genomic-environment sources of G × E in our tropical germplasm. For example, Vidotti et al. (2019) used GWAS to establish a relation between the genetic control of the maize responsiveness and *Azospirillum brasilense*, a plant growth-promoting bacteria (PGPB) common in tropical soils and related to maize nitrogen fixation. The GWAS outcomes helped understand how heterosis is important for improving the quality of crop systems by increasing the nitrogen use efficiency (NUE) of maize. Another promising approach is presented by Millet et al. (2016), which involves the use of GWAS to find genomic regions associated with the reaction norm for key environmental factors expected in future scenarios.

A similar approach uses only the phenotypic data to model parameters of adaptability and stability, as in the work by Gage et al. (2017). Combining GWAS and such parameters that reflect the effect of G × E for specific genotypes, these authors were able to explore the genomic-related sources that explain the drivers of phenotypic plasticity and how the artificial selection shaped these patterns on the temperate maize germplasm in the United States. Finally, another good example is given by Ramstein et al. (2020). These authors used GWAS to find quantitative trait loci (QTLs) related to the phenotypic variation of some important traits in maize. Then, using gene annotation, it was possible to explore the functional contribution of those QLTs to express the phenotypes and the increasing accuracy of GP. This functional enrichment in further GP models contributed to the increase in the accuracy of a hybrid panel of temperate maize cost-effectively. It can also be useful for the tropical germplasm, which still demands the development of a higher panel of inbred lines to address the test of those hypotheses.

### How to Deal With the Complexity and Diversity of Big Data?

In the last 20 years of genomic selection research, the plant breeding community is still learning how to connect a wide number of data sources related to the “Central Dogma of Molecular Biology” with the observed phenotypic variation of traits in field trials, which began as a regression of phenotypes over molecular markers evolved by the integration of different data sources and modeling structures. Computational research in GP must develop to capture other data sources in a computationally smart way and find which structure is better to integrate each type of data. For example, Costa-Neto et al. (2020) suggest that the use of Deep Kernels (DK) is a faster and more accurate way to model both genomic and enviromic relatedness than benchmark GBLUP approaches, which is similar to results by Cuevas et al. (2019) who used near-infrared data. However, it seems that the paradigm of “less means more” when dealing with some sources of data, such as enviromics, in which we still have a long pathway in optimizing approaches capable of capturing gene × envirotype interactions across crop fields. In addition, in our studies, we observed that a good enviromic kernel (W) added in the GP models as the main effect is sometimes better than modeling a full-rank reaction-norm model accounting for the genomic environment and genomic enviromics. On the other hand, works by authors, such as Cuevas et al. (2020) and de los Campos et al. (2020) show that big historical data can be implemented by different computational approaches and have a satisfactory accuracy to support the selection decisions. Thus, methodological approaches must be developed to capture exploitable patterns in big data and computational tools to implement them, the latter preferably as open-source software.

Deep learning approaches accounting for this data source can be a more parsimonious approach to taking advantage of big data without over-fitting prediction models. Finally, we find that using multi-trait multi-environment data might help design better field phenotyping trials for training GP models. As the modern computational tools attempt better to explore G × E and G × G within a multi-environment multi-trait context, the opposite path might be taken by using historical data to design future trials (Rincent et al., 2017) and scenarios (Millet et al., 2016; Bustos-Korts et al., 2019), but also to predict cultivars at novel growing conditions (Gillberg et al., 2019; Millet et al., 2019; de los Campos et al., 2020).

### Are Prediction-Based Tools Cost-Effective Approaches?

Prediction-based tools are cost-effective approaches. Plant breeding is based on selecting the best-evaluated genotypes in target environments, demanding many field-testing resources (physical and financial). Therefore, GP has proven to be useful to enlarge the spectrum of individuals evaluated *in silico* but with a limited accuracy in multiple environmental conditions due to the non-additive effects related to G × E and G × G interactions. Recently the emerging new ways to include environmental data and CGM in the GP are considered good strategies to correct this deficiency in predicting G × E interaction deviations (Messina et al., 2018). In addition, these new applications allow genotype screening at reduced phenotyping costs considering virtual scenarios.

Despite the great advances that have been made, what is to come is exciting for hybrid maize breeding. New tools and models, such as the integrated use of high throughput phenotyping, CGM, and optimized tools for simulation of improvement methods can bring more resolution, realism, and depth to the predictions. With HTP, we will be able to evaluate the same plant several times over the crop cycle and increase the effective size of training populations. Additionally, even before running HTP studies in the field, it is possible to validate some protocols *in silico* for phenotyping traits, such as PH (Galli et al., 2021). On the other hand, both pathways of enviromics and CGM will allow us to build virtual improvement scenarios and predict the deviations of G × E interaction more accurately. Finally, with the simulations, we will be able to test a series of scenarios cheaply and easily, helping outline the best improvement strategies and resource allocations.

### Finding Research Partnerships to Expand the Field-Testing Network

Most of the applications described in the last section consider datasets with at least four environments and almost one thousand entries (lines, DH, and hybrids), which represent the reality for at least a small-scale breeding program. As discussed in the previous sections, with the increase in the availability of data, the computational demand and the power of cutting-edge testing hypotheses in maize breeding also increase (Rogers et al., 2021). We envisage that maize hybrid breeding programs can take advantage of historical multi-environment testing data (Dawson et al., 2013) to explore the environmental impacts on the plasticity of germplasm, collecting during this process data from enviromics, and other sources of data useful to train accurate models. During this step, it is possible to integrate some simulation platform capable of generating reliable environmental scenarios (Millet et al., 2016) or phenotypic landscapes (Bustos-Korts et al., 2019), such as CGM. The use of public databases to test hypotheses, train models, or import datasets for your own purposes that might reduce costs and provide a guideline to follow. However, as we have pointed out in section Germplasm Characterization, the implementation of a well-conducted field trial for phenotypic, genotypic, and envirotypic characterization of the so-called “Modern Plant Breeding Triangle” (Crossa et al., 2021), is crucial for providing good quality data to test a wide number of hypotheses. Another interesting option is to establish partnerships with other small-scale breeding programs and public institutions in order to create a large network of field data, such as the successful partnership of public institutions in the United States—*The Genome to Field Project* (McFarland et al., 2020). In Brazil, the first steps of this approach were led by the Allogamous Plant Breeding Laboratory from USP. We tried to share every genomics database, enviromics, and high-throughput phenotyping (available in https://data.mendeley.com/datasets/5gvznd2b3n).

## Data Availability Statement

Publicly available datasets were analyzed in this study. This data can be found here: https://data.mendeley.com/research-data/?page=0&search=%22Roberto%20Fritsche%20Neto%22.

## Author Contributions

RF-N conceived and designed all studies. GG, FA, FS, DL, PM, LB, GC-N, and IG generated the dataset and performed the data analysis. RF-N wrote the manuscript. GG, KB, GC-N, and JC revised the text, which all the authors finally edited. All authors contributed to the article and approved the submitted version.

## Conflict of Interest

The authors declare that the research was conducted in the absence of any commercial or financial relationships that could be construed as a potential conflict of interest. The handling editor declared a past co-authorship with one of the authors JC.
